# Shifts in learning assistants’ self-determination due to COVID-19 disruptions in Calculus II course delivery

**DOI:** 10.1186/s40594-021-00312-0

**Published:** 2021-10-16

**Authors:** R. L. Hite, G. Childers, J. Gottlieb, R. Velasco, L. Johnson, G. B. Williams, K. Griffith, J. Dwyer

**Affiliations:** 1 College of Education, Texas Tech University, 3002 18th Street, Lubbock, TX 79409 USA; 2 College of Education, The University of Iowa, 240 South Madison Street, Iowa City, IA 52242 USA; 3 Center for Transformative Undergraduate Experiences, Texas Tech University, Drane Hall #239, MS 1010, Lubbock, TX 79409 USA; 4 College of Arts and Sciences, Texas Tech University, P.O. Box 41034, Lubbock, TX 79409 USA; 5 STEM Teaching, Engagement and Pedagogy (STEP), Texas Tech University, P. O. Box 43131, Lubbock, TX 79409 USA

**Keywords:** Calculus, COVID-19, Learning assistant, Online learning, Self-determination theory, Undergraduate STEM

## Abstract

**Background:**

The Learning Assistant (LA) model with its subsequent support and training has evidenced significant gains for undergraduate STEM learning and persistence, especially in high-stakes courses like Calculus. Yet, when a swift and unexpected transition occurs from face-to-face to online, remote learning of the LA environment, it is unknown how LAs are able to maintain their motivation (competence, autonomy, and relatedness), adapt to these new challenges, and sustain their student-centered efforts. This study used Self-Determination Theory (SDT) to model theoretical aspects of LAs’ motivations (persistence and performance) both before and after changes were made in delivery of a Calculus II course at Texas Tech University due to COVID-19 interruptions.

**Results:**

Analysis of weekly written reflections, a focus group session, and a post-course questionnaire of 13 Calculus II LAs throughout Spring semester of 2020 showed that LAs’ reports of competence proportionally decreased when they transitioned online, which was followed by a moderate proportional increase in reports of autonomy (actions they took to adapt to distance instruction) and a dramatic proportional increase in reports of relatedness (to build structures for maintaining communication and building community with undergraduate students).

**Conclusions:**

Relatedness emerged as the most salient factor from SDT to maintain LA self-determination due to the COVID-19 facilitated interruption to course delivery in a high-stakes undergraduate STEM course. Given that online learning continues during the pandemic and is likely to continue after, this research provides an understanding to how LAs responded to this event and the mounting importance of relatedness when LAs are working with undergraduate STEM learners. Programmatic recommendations are given for enhancing LA preparation including selecting LAs for autonomy and relatedness factors (in addition to competence), modeling mentoring for remote learners, and coaching in best practices for online instruction.

## Introduction

Research-oriented institutions offer large lecture sections of introductory level science, technology, engineering, and mathematics (STEM) courses, which structurally reduce interactions between instructors and students (Geske, 1992). Yet, interpersonal interactions with instructors/professors are vital for undergraduates to understand STEM content (Jardine et al., 2020), enhance course satisfaction (Cuseo, 2007), and increase retention in STEM majors (Alzen et al., 2018). Positive interpersonal interactions have been found critical in recruiting and retaining students identified by the National Science Foundation (NSF) as under-represented minorities (i.e., non-white heterosexual males) in STEM fields (Cole, 2010; Cole & Espinoza, 2008; Cole & Griffin, 2013; Kim & Sax, 2009); notably, this “contact with faculty has been linked with increased student success, particularly among under-represented students, as students who interact more frequently with faculty tend to earn higher grades, increase their likelihood of degree completion, and increase their degree aspirations” (Hurtado et al., 2011, p. 554). Without this interaction, STEM courses can seem ‘chilly,’ leaving students to feel isolated and doubting their belonging in STEM (Lichtenstein et al., 2014), especially among women and minority students (Chelberg & Bosman, 2019). However, it is infeasible for the faculty member to interact with each student or groups of students within the lecture period to foster this support for content mastery and personable interaction. One adaptive strategy to address the dearth of lecture-embedded interpersonal interactions to support STEM students is the use of *learning assistants* (LAs). LAs are undergraduate students who were recent and successful participants in undergraduate STEM courses. As an LA, they are coached in both content and pedagogy, attended the lecture meetings of STEM courses (such as Calculus II), and during lecture, provided in-class support to pre-assigned groups of students (Learning Assistance Alliance [LAA], 2020a). Ascribed as ‘near-peers,’ LAs have recently mastered the course content and are able to empathize with challenges that current students can have in the STEM course (Otero, 2015; Otero et al., 2006). The three-pronged LA model (LAA, 2020d) prescribes means to facilitate student-centered learning in STEM higher education by (1) planning upcoming content with the course (content) instructor; (2) learning and reflecting upon weekly best practices with a pedagogical mentor; and (3) interacting with groups of students by facilitating one-on-one or small group discourse in each lecture period.

Research on LA programs suggests STEM students, especially minority students (Van Dusen & Nissen, 2020), experience greater satisfaction (Talbot et al., 2015; Thompson & Garik, 2015) and achievement (Sellami et al., 2017) in large lecture-based, introductory STEM courses supported by LAs. Notably, research on LAs focuses on STEM student outcomes, suggesting that this success rests on LAs’ abilities to engage successfully in practice, content, and pedagogy (LAA, 2020d), which may ultimately lie in their self-determination or motivation. The Center for Determination Theory (2021) defines self-determination as one’s abilities to engage in specific work (*persistence*) and how well they engage in that specific work (*performance*). Both persistence and performance are important factors in ensuring the longevity and efficacy of LAs in their work supporting STEM learners.

Given the $750 compensation for serving as an LA per semester and the required, rigorous classroom duties to receive a single hour of an elective undergraduate math credit, we contend that the students who apply to and are selected for LAs programs are attracted to the intrinsic attributes and benefits of the position (e.g., genuine interest in teaching, a desire to help others, or enjoyment of the course content). Thus, we posit the lens of SDT is ideal in viewing how motivational shifts occur when their expectations of that work changes rapidly. To that end, we explored shifts in LAs’ self-determination in a face-to-face (F2F) section of Calculus II using the three constructs of Self-Determination Theory (SDT). We used both their written and spoken perceptions competency, relatedness and autonomy throughout the COVID-19 facilitated disruptions to course delivery in Spring of 2020 at Texas Tech University. COVID-19 policy restrictions shifted this course to a remote modality, abruptly transitioning faculty, undergraduates, and LAs to distance (remote, online) instruction halfway through Spring semester of 2020. Using qualitative data sourced from 13 LAs in Calculus II in spring of 2020, this research study describes LAs’ self-determination from before and after COVID-19 interruptions in course delivery modality. Previous scholarship on LAs has explored self-efficacy and discussed links to motivation as being salient to their effectiveness to their work now and into the future (Cao et al., 2018; Fineus & Fernandez, 2013; Jakyma, 2017; Kayes et al., 2014). Furthermore, individuals who have psychological needs met become motivated and will “voluntarily and sincerely provide their best abilities in carrying out their duties and responsibilities” (Kuswati, 2019, p. 283), suggesting a positive relationship between motivation and effectiveness. To extend this literature, we have chosen to explore the nature of their motivation by disaggregating and examining the three factors of motivation (i.e., competence, autonomy, and relatedness), ascribed by Self-Determination Theory (Ryan & Deci, 2000a, 2000b). Through SDT, we can measure and model how their self-determination was impacted, such that when similar or continued interruptions occur that influence LAs’ self-determination. Notably, these three constructs of SDT have been collectively (Marshik et al., 2017) and individually related to effectiveness as described in teacher education literature for competence (e.g., Okoli, 2017), autonomy (e.g., Nguyen et al., 2021) and relatedness (e.g., Guay et al., 2019). This study was guided by these research questions: (1) In what way were LA’s perceptions of self-determination, evidenced by the constructs of competence, autonomy, and relatedness during face-to-face instruction and (2) In what ways did those constructs shift with the rapid transition from face-to-face online learning due to COVID-19 interruptions? By examining the constructs individually, we better visualize aspects (i.e., competency, autonomy, and relatedness) that contribute to LAs’ motivation and performance. This information is helpful in identifying and thus, addressing needs of LAs’ during times of stress and/or unexpected transition in the future, such as to bolster their persistence and performance in supporting undergraduate STEM students.

## Background

LAs are undergraduate students who serve as group learning facilitators for other undergraduate students (LAA, 2020c). LAs receive content support, usually from the course instructor, while also completing a special LA pedagogy course from a pedagogical mentor (LAA, 2020c). Originally developed at the University of Colorado Boulder as an intervention to simultaneously enhance the quality of undergraduate science instruction and recruit talented science students into K-12 teaching (Otero et al., 2006), LA programs have grown significantly in scale and diversity. The Learning Assistant Alliance (LAA), hosted by the University of Colorado Boulder, reported 481 member institutions and more than 200 active LA programs around the globe (LAA, 2020b). The LA model has been extended outside of the sciences, but the majority of programs remain in STEM disciplines (Thompson et al., 2020). Despite this growth and adaptation to new undergraduate contexts, the LA program remains mostly consistent.

There is growing evidence that LA programs enhance student outcomes in supported courses and even subsequent courses (Alzen et al., 2018; Barrasso & Spilios, 2021; Pollock, 2009; Talbot et al., 2015). LA programs appear to foster positive subject-matter mastery and identity formation among the LAs themselves (Close et al., 2016; Davenport et al., 2017). While more research is still needed, evidence continues to grow to support claims that LA programs can enhance K-12 teacher recruitment (Otero et al., 2010) and training (Gray et al., 2016; Thompson et al., 2020). Current efforts to increase the number and diversity of STEM graduates in the United States provide a strong rationale for investing energy and resources in supporting better student outcomes in STEM undergraduate courses. Despite ambitious national calls to significantly increase the number of STEM graduates (President’s Council of Advisors on Science and Technology [PCAST], 2012), the United States continues to face a significant need to “strengthen, grow, and diversify its science, technology, engineering, and mathematics (STEM) workforce” (PCAST, 2020, p. x). As a traditional gateway course sequence to STEM degree completion and subsequent career attainment (Nelson, 2010; Rasmussen & Ellis, 2013), calculus is of particular importance in this work. Success in calculus corresponds with successful completion of a STEM degree, and students who experience difficulties in learning calculus increase their chances of leaving a STEM degree plan (Chen, 2013).

This research builds on three components of the emerging LA model literature. First, this project adds to the general body of research by examining a relatively understudied group in LA programmatic literature, which are the experiences of the LAs themselves in supporting undergraduate STEM learners (Close et al., 2016; Hite et al., 2021; Top et al., 2018). Second, we use a vetted lens, Self-Determination Theory, to model and understand how LAs were motivated in their growth and abilities to serve undergraduate STEM learners in a period of rapid change and transition in their professional modality of serving those learners. And third, this article directly addresses the real-time challenges in supporting undergraduate STEM education (Forakis et al., 2020; Griffiths, 2020), through LA programs (Emenike et al., 2020; Gemmel et al., 2020), during the current challenge of the COVID-19 pandemic and for future interruptions to come.

### Theoretical framing

Self-Determination Theory (SDT) was utilized as the theoretical framework for this study to provide a lens in documenting LAs’ motivation to support students in a Calculus II university class. SDT posits that a person’s motivation, social functioning, and personal well-being are related to the extent in which a person’s choices are self-determined and an individual’s behavior is aligned with that person’s sense of self (Deci & Ryan, 1985, 2002; Ryan & Deci, 2000a, 2000b). In turn, self-determination has been linked with intrinsic motivation as well as extrinsically motivated behaviors such as self-regulation (Ryan & Deci, 2000b).

Ryan and Deci (2017) have identified innate psychological needs that are related to motivation via self-determination; these needs are competency, autonomy, and relatedness. First, competency is defined as a sense of confidence and efficacy in one’s actions (Deci & Ryan, 2002). Second, autonomy is the extent to which an individual perceives their actions as originating within themselves, even if an action is at the request of others. Third, relatedness is a sense of connection and caring relationships with others, especially those in one's community. It is noted that, “this final need, though often overlooked, is the most significant of the three because if a person’s need for relatedness is not fulfilled, that person is unlikely to engage in the activities and relations that would lead to fulfillment of the other two needs” (Darner, 2009, p. 45). Together, these constructs describe the psychological needs that need to be met for an individual’s choice to be felt as self-determined, and thus for an individual to feel that they are acting in a way that is aligned with his or her sense of self or purpose. Any change among the three constructs can quantify and qualify how motivation (i.e., perceptions of competency, autonomy and relatedness) has varied and impacted the individual’s “performance and persistence” (Center for Self-Determination Theory, 2021, para. 14).

SDT has been applied in a wide range of studies in education to explore the relationship between competence, autonomy, relatedness, and motivation (Chen & Jang, 2010; León et al., 2015; Sørebø et al., 2009; Standage et al., 2005). Among the LA literature and related undergraduate teaching assistant literature, motivation has been a salient and recurring theme (e.g., Cao et al., 2018; Filz & Gurung, 2013; Jakyma, 2017; Kayes et al., 2014). One study by Fineus and Fernandez (2013) found intrinsic motivation to be an important factor for a Calculus LA to become a mathematics teacher.Further, SDT has been used as a model in two different awarded proposals within the Robert Noyce Teacher Scholarship Program (e.g., NSF ) to better understand how LAs uniquely learn the skills of teaching through their positions serving students and its relationship to persistence in pursuing K-12 teaching futures. We believe the use of SDT in this study is a logical extension of the literature on LAs as a well-established and appropriate choice for this project because this theory describes (through specific constructs) how individuals develop and maintain the motivation and self-regulation needed to persist in a given task, such as being an LA and during a period of rapid transition like exploring the impact of the COVID-19 interruptions on LAs. SDT provides a way to understand how some of the LAs’ basic psychological needs, such as competence, autonomy, and relatedness, changed as the university abruptly transitioned online in March of 2020, and how the changes in those needs may have impacted their self-determination (persistence and performance) as LAs.

## Methods

To investigate how the COVID-19 interruption (moving Calculus II classes from F2F to online) had impacted LAs’ effectiveness through the lens of self-determination, a deductive qualitative content analysis was employed to explore the frequency and fluctuations of the three constructs of SDT within the COVID-19 facilitated event. By using qualitative content analysis, we mined rich qualitative data from several sources (e.g., students’ journals entries, focus group, questionnaires), to document and chronicle how the COVID-19 facilitated interruption in teaching modality impacted LA’s self-reported aspects of self-determination through the theoretical SDT constructs of competence, autonomy, and relatedness. Using SDT constructs as the a priori coding schema not only provided a vetted means to model for LAs’ self-determination, but also highlighted how shifts within their self-determination occurred over time, punctuated by the rapid transition from F2F to remote learning. In other words, as LAs reported their experiences, we could categorize those experiences into the three SDT constructs to better understand how their experiences were impacting their self-determination as an LA during these time periods. Reporting of results using qualitative content analysis allowed for ascribing meaning to the categories and their frequency vis-à-vis the theoretical model chosen (Elo & Kyngäs, 2008), which had made qualitative content analysis a popular yet robust method in the social (White & Marsh, 2006) and medical (Forman & Damschroder, 2007) sciences. This research was reviewed and approved by the University’s Institutional Review Board (IRB) under IRB2020-373 (for reflection data) and IRB2020-625 (for focus group and questionnaire data).

### Participants

Participants included all 13 of the LAs who were part of the Learning Assistant Program in the large, introductory section of Calculus II in Spring of 2020. The LA pool consisted of self-selected, upper-level undergraduate STEM students who had a cumulative Grade Point Average of 3.0 or higher, earned an A or B in the specific STEM course for which they would serve as an LA, had an interest in supporting undergraduate STEM learning, and would like to receive an undergraduate math credit for their service. Because students self-select into the LA program, all students who met the criterion (i.e., had previously taken the course with final grade of B or higher) were accepted into the LA program. Most students (12 out of the 13) accepted into the LA program were eligible (i.e., U.S. citizens) to be part of a Noyce grant at the University, in which they also received a stipend of no more than $750 per semester. Notably, there were no specific recruitment activities for women or racial/ethnic minorities (i.e., non-white) in the LA program as the LA program is advertised equally among the undergraduate STEM courses in spring and fall terms.

Each LA had an equivalent amount of LA experience since all were new to the program. Each LA was given a pseudonym and are listed here in alphabetical order with their self-ascribed race/ethnicity and gender identification: Alejandra (Hispanic female), Arturo (Hispanic male), Chloe (White female), Connor (White male), Dylan (White male), Eduardo (Hispanic male), Jake (White male), Josefina (Hispanic female), Lucas (White male), Maria (Hispanic female), Rachel (White female), Ramesh (South East Asian male), and Rashidi (African male).

### Setting/context

Adapted from the Colorado Learning Assistant model from the University of Colorado in Boulder (Otero et al., 2010), expectations of the LAs at Texas Tech University were to: (1) attend and serve an assigned group of 10 undergraduate students enrolled in a Calculus II course (3 h per week); (2) attend LA preparation sessions (2 h per week) with LA faculty mentors; (3) be available to meet with their respective course students (1 h per week); and (4) reflect upon their LA experiences each week as a part of their programmatic experiences where they would write about elements of content, pedagogy, and practice (LAA, 2020d). Each of these four elements are explained as follows:

1. During class, LAs were expected to be with their student groups during lecture and available to answer questions as they arose during lecture and help facilitate small group discussion during lecture. Prior to COVID-19 interruptions, these interactions occurred face to face within the lecture hall. With the transition to online learning, all lecture-based interactions occurred via Zoom by using the chat with students during lecture and utilizing smaller break-out rooms for small group interactions.
2. Each week, before the lecture sessions, LAs met and were mentored by a content (Calculus II) and a pedagogy (teaching) professor. In Spring of 2020, the content mentor was a full professor in mathematics and the instructor of record for the Calculus II course. The pedagogy mentor was a STEM faculty member and director of the LA program at the university. In each session, the content mentor discusses the information that will be presented and activities that will occur during lecture, highlighting areas in which students may encounter difficulties in comprehending the content. The pedagogical mentor supplements the content mentor’s presentation with pedagogical tips to engage STEM learners vis-à-vis the content presented. The pedagogical mentor also addresses questions and concerns the LAs raise either during this meeting or from their written reflections. These meetings occurred face to face prior to the COVID-19 interruption, and then subsequently over Zoom. These weekly meetings are part of their LA course; each participating student receives credit for an undergraduate math course in their respective degree plans as an LA.
3. Each LA held office hours for their small group of students each week. Prior to COVID-19 interruptions, office hours occurred face-to-face and then transitioned online to Zoom.
4. The purpose of reflection journals was not only to adhere to the LA model of weekly reflection, but also serve as data for program evaluation and NSF Noyce grant reporting. Participating faculty reviewed the students’ reflections to help inform the topics (content and pedagogy) for the following week of mentoring sessions. Students received part of their math credit for submitting reflections as well as helping students during lecture, attending the LA meetings and holding office hours. Therefore, all feedback from their journal entries were given orally and targeted to the group (unless an LA had a specific question or issue) during the weekly group meetings with faculty mentors. Hence, LAs were not assigned a word count for each journal reflection, but were asked to write at least a half-page reflection each week.

These activities occurred through the 15 week semester of Spring 2020, which was held F2F from January 15, 2020, to April 3rd, 2020. After the first of April 2020, all interactions with LAs, faculty and Calculus II students were remote (e.g., meetings via email) and virtual (e.g., instruction over Zoom) until May 12, 2020, due to university-level policy changes regarding COVID-19.

### Positionality

The first five authors were most involved in the cleaning, analysis and write-up of the data received in the study. These individuals however played no role in the day-to-day functioning of the LA program at Texas Tech University. These authors had knowledge of the LA program through the larger Noyce grant (either as Co-PI or senior personnel) and had skills in qualitative research to implement and analyze the present research study. However, the latter three authors served as the content and pedagogical mentors of Calculus II LAs and the PI of the Noyce grant at the institution, respectively. These individuals were instrumental in the systematic collection of data that was analyzed and interpreted by the former authors.

### Data

Three sources of qualitative data were collected for the descriptive case at various time points in the study. The primary source of data were LAs’ journal entries (*N* = 70, *M* = 5, SD = 1.5). Each journal entry consisted of a detailed narrative response, due each Friday during the spring semester of 2020, in which LAs independently and weekly reflected upon their successes and challenges as a Calculus II LA. The open-ended prompts for each entry were as follows: (1) What is working well? (2) What is not working well? (3) What suggestions do you have? After March of 2020, program faculty wanted to elicit information to how LAs were feeling and how they could better help them to be successful in this new class delivery format. Given that the faculty’s concern addressed LA’s psychological needs (tied to motivation), as a result, this additional prompt about their successes and challenges during and after the transition provided more data in how LAs were effective in supporting students, having agency to perform their function as an LA, and feelings of relatedness to their STEM learners once transitioning from face-to-face to the online modality was added. LAs were not intentionally introduced to SDT; however, the new reflection task had elements of the three constructs of SDT embedded into the prompts (i.e., asking their perceptions of competency, relatedness, and autonomy as an LA in the new online environment). The average length of the journal entries was 263 words (median = 176, SD = 232), with a maximum length of 1235 words and a minimum length of 9 words. Journal entries were parsed by reflections completed before (*n* = 39, February to March) and after (*n* = 31, April to May) the transition to remote instruction. Each journal entry was collected, de-identified with a pseudonym, and coded via SDT constructs to capture current levels and shifts in self-determination of LAs, prior to and after remote learning. The only LA who did not provide any reflections after the transition was Dylan, although he remained as a participating LA in the program through May. All reflections, collected via email, were loaded into a spreadsheet and de-identified for content analysis.

In June, one month after the end of spring semester, all LAs were invited to participate in a 60-min focus group conducted via Zoom that was audio recorded. Three LAs (Lucas, Josefina, and Eduardo) were available to engage in a conversation, facilitated by a researcher, reflecting upon their experiences as an LA during three discrete time points in the semester: before, during, and after the course modality transition due to COVID-19. Prompts of the focus group protocol had LAs consider and reflect upon experiences in Spring 2020 in which they felt in/competent, not/autonomous and un/related to their Calculus II students and one another. The interviewer was a recent graduate of the university having received their doctoral degree in education (non-STEM). This person had no prior or current relationship to the LAs or the LA program at the university to mitigate bias in LAs’ responses. All captured LA focus group responses were de-identified to their pseudonym and promptly coded to SDT categories to provide greater understanding of SDT construct frequencies.

In August, 3 months after the end of spring semester, LAs were provided an online open-ended questionnaire, via Qualtrics, tasking them to contemplate more holistically upon their experiences as an LA. All but three LAs (Alejandra, Arturo, and Connor) responded to the questionnaire. The six prompts were: What have you learned from working with faculty and other LAs?; What have you learned from working with undergraduates?; What were challenges that you did not originally anticipate as an LA?; How has your work as an LA connected to the (Calculus II) material?; In what ways, if any, has your work as an LA related to your career plans?; What other knowledge, skills, or dispositions have you garnered from being an LA? Participants provided their answers in separate text boxes in the questionnaire, which yielded 60 responses in total. The responses were de-identified to their corresponding pseudonym and loaded into a spreadsheet for content analysis.

### Analysis

To produce a multi-grained understanding in the fluctuation of self-determination theoretical constructs, among and within LAs, both prior to and after the COVID-19 facilitated transition from F2F to online learning in Calculus II a qualitative content analysis (per Krippendorff, 1980) was performed on all three qualitative data sources. Qualitative content analysis “is a powerful [analytical] method…[and] a very powerful visualization tool” especially when it comes to analyzing large amounts of qualitative data (Schreier, 2012, p. 37, 256). Operationally, each of the three SDT constructs were used deductively to describe LA’s changes in self-determination since all three constructs contribute to one’s performance and persistence (Center for Self-Determination Theory, 2021) as self-determination. Using SDT, we may model how each construct manifests (within construct categories) and to what amount (via frequencies), both before and after the course delivery change in modality. Naturally, frequency counts by constructs only provide a quantitative measure of change occurring in constructs over time. Therefore, we also include *qualitative* explanations (quotations) that the LAs have written or said to provide the insight needed to understand their shifts in self-determination (in performance and persistence as an LA) when serving STEM learners during this time of transition. Notably, *qualitative* content analysis differs from *quantitative* content analysis because the analysis did not focus on locating and quantifying specific words, which warrants a degree of making hypotheses on the data itself. In a qualitative content analysis, we may use extant SDT theory, operationalized through the three constructs, to code the data (rather than just a basic quantitative querying process) and to visualize construct frequencies (rather than just discussing findings holistically) (see Schreier et al., 2019).

**Fig. 1:**
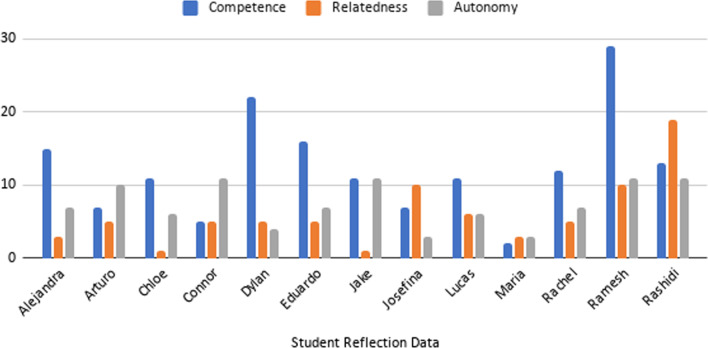
Frequency counts of SDT constructs before the transition to remote learning

**Fig. 2:**
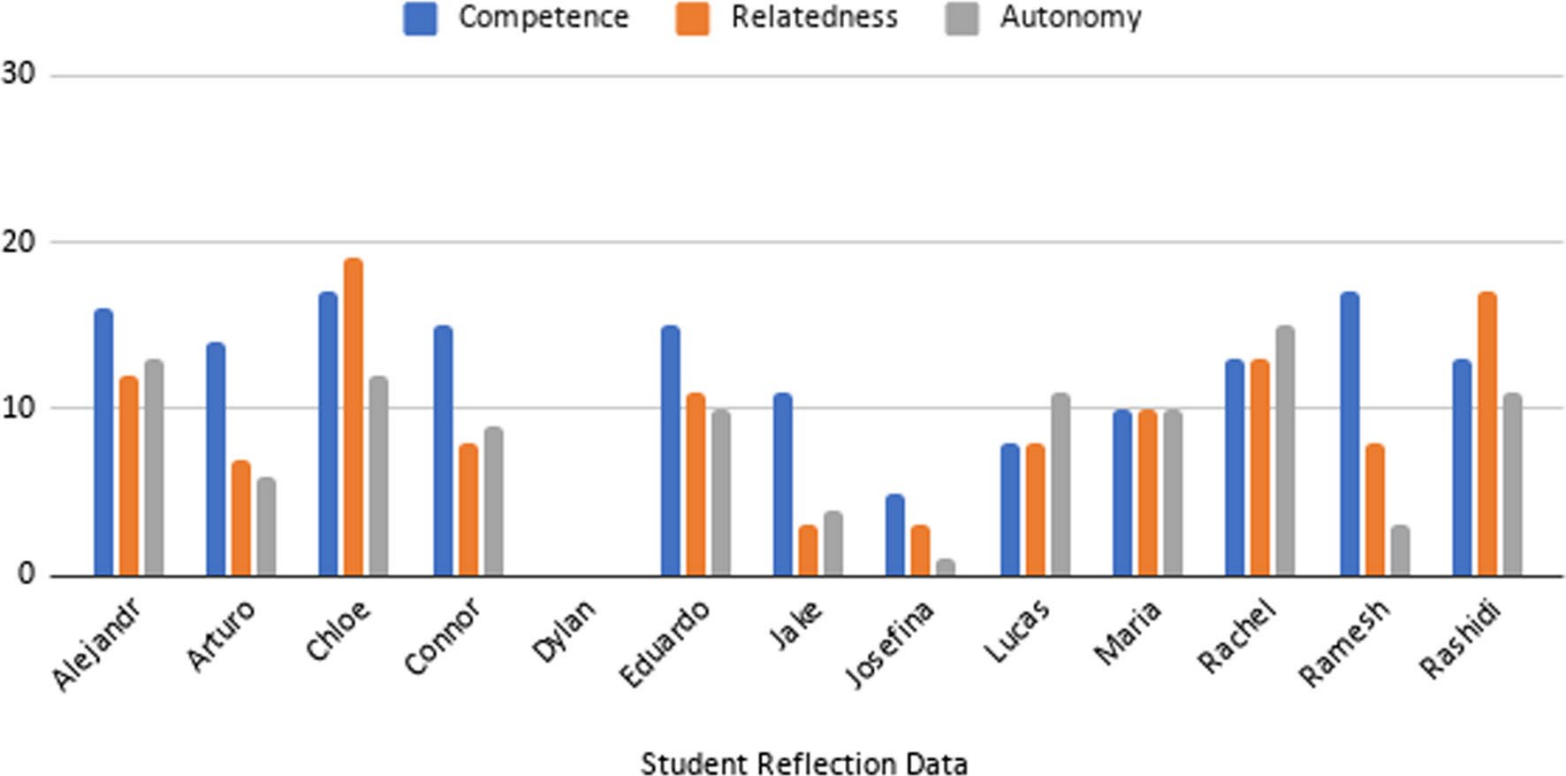
Frequency counts of SDT constructs after the transition to remote learning

To conduct this process, first, each transcript (journal entries, focus group discussion, and questionnaire responses) was reviewed (first pass) to produce units of data that were related to SDT. In the second pass, these units of data were coded to one of the three constructs of SDT. In sum, 714 units of data were coded and sourced from the 70 journal entries (yielding an average (mean) of 55 coded units of data per LA (Median = 53, SD = 17). From the questionnaire, we sourced 87 coded units of data for an average of 9 coded units of data (Median = 9, SD = 1.4) from each of the 10 participants’ responses to the six question prompts. The focus group, held with just three participants, yielded 10 coded units of data for an average of three unique contributions per LA to SDT coding (Median = 3, SD = 0.6). From the analysis, we developed tables to evidence how we coded our data to the SDT framework (Table 1) and by data type (Tables 2, 3, and 4, respectively). We also calculated frequencies to demonstrate how constructs are proportionally represented in the data set (Figs. 1, 2) within the time period of transition. We report how constructs shifted using coded qualitative data in a descriptive narrative, going construct-by-construct. This methodological process, including how we defined constructs of SDT and how we coded data by SDT constructs, is aligned to other studies using SDT to explore aspects of motivation in teaching and learning contexts (e.g., Hite et al., 2019; Jacobi, 2018; Trenshaw et al., 2016; Virkkula, 2020).

**Table 1:** Self-Determination Theory codebook with sample quotations from journal entries

SDT construct	Explanations of SDT Construct	Coded quotations from reflection data	Audit trail
Competence	Outward displays		
Understanding their role as LA (performance of certain tasks)		*We have kind of established this rhythm as we LA’s know our roles*	Rashidi 2-14-2020
Perceptions/observations of being capable as an LA		*I think, personally, I just do better in a face-to-face setting*	Josefina 3-14-2020
Desire or motivation to be capable as an LA		*Most of us had the technology available to communicate effectively and were motivated to do so*	Connor 5-2-2020
Actions made to improve content knowledge or skills; NOTE: not relationships		*I can confidently say that explaining the intuition behind the formula for integration by parts helps tremendously*	Jake 2-13-2020
	“I did, I tried…” statements (Action verbs)	*I couldn’t come up with a way to not give them the answer without stressing them out to give up on the answer*	Eduardo 2-21-2020
Autonomy	Inward reflections		
Recommendations for current or future practice		*I don’t know if it would be feasible to do worksheets in the remaining lessons, but maybe some kind of class practice might help*	Rachel 3-2-2020
Making decisions on building relationships; NOTE: not directly tied to relatedness		*I plan to focus more on giving 'small hoorahs’ for completing the harder steps in integrals as a confidence boost, and researching on how to aid re-instillation of faith in a student*	Arturo 2-17-2020
Making decisions on content/pedagogy; NOTE: not directly tied to competence		*Online communication is great, but in my opinion face to face is better*	Lucas 5-4-2020
Plans made to improve upon current for future performance as LA (Being an LA in the future)		*I think I’m a fairly okay instructor right now, but I know I have a lot of room to improve and I make mistakes*	Dylan 2-28-2020
	“I believe/think I planned” statements	*I definitely think that the level of enthusiasm decreased with the switch to online learning, due to the fact that students were sent home, which is an environment that they usually only go back to for breaks/summers*	Chloe 5-2-2020
Relatedness	Personal interactions		
Assessments/ Observations made about relationships with students (feelings to action)		*Remembering them, and their quirks. Knowing them as people really helps me to know when I need to assist them in math, I feel*	Ramesh 2-17-2020
Assessments/ Observations made about relationships with mentors		*[I have learned lots from] Mr. G pedagogy meetings over instructional content*	Alejandra 5-5-2020
Assessments/ Observations made about relationships with other LAs		*I think that the entire room is our domain, and we should not be down on ourselves because someone else is helping our row*	Chloe 2-28-2020
Actions (or inactions) made to improve/erode relationships; NOTE not content knowledge or skills		*The large disconnect from not seeing everyone again kinda shattered the camaraderie that was present in my row*	Lucas 5-4-2020
“I felt… I noticed…” statements		*It took a week or two at the beginning of the semester in order for students to first become comfortable enough to approach me whenever they had a question*	Maria 5-5-2020

**Table 2:** Supporting quotations from LAs’ reflections to the three constructs of SDT before, during and after the remote learning transition

Construct	Before remote learning experience	During and after remote learning experience
Competence	“I can confidently say that explaining the intuition behind the formula for integration by parts helps tremendously.” (Jake)	“Most of us had the technology available to communicate effectively and were motivated to do so.” (Connor)
Autonomy	“I plan to focus more on giving ‘small hoorahs’ for completing the harder steps in integrals as a confidence boost and researching on how to aid re-instillation of faith in a student.” (Arturo)	“I definitely think that the level of enthusiasm decreased with the switch to online learning, due to the fact that students were sent home, which is an environment that they usually only go back to for breaks/summers.” (Chloe)
Relatedness	“Remembering them, and their quirks. Knowing them as people really helps me to know when I need to assist them in math, I feel.” (Ramesh)	“The large disconnect from not seeing everyone again kind of shattered the camaraderie that was present in my row.” (Lucas)

**Table 3:** All quotes coded from LA focus group to the three constructs of SDT before, during, and after the remote learning transition

Construct description	Before remote learning experience	During and after remote learning experience
Competence experiences making you feel more competent or successful	“…being able to see [the students] work it out and do it.” (Eduardo)	“I just use the white board on zoom…whenever I had people [to] engage…” (Josefina) “I felt most confident in my [online] breakout rooms.” (Lucas)
Competence experiences making you feel less competent or successful	“Sometimes I would have a misconception and thought I was teaching them something right.” (Lucas)	“I lost the respect. Whenever it switched on the breakout room. We would often get derailed.” (Lucas) “There was definitely a learning curve.” (Eduardo)
Autonomy being empowered to make decisions regarding content, pedagogy, and forging relationships with undergraduate students	“Whenever we would go over a concept, some of the methods and the things that, each of us were using our [own] methods.” (Eduardo)	“Having the technology would be important. Sometimes I would use the whiteboard. So how to engage my students not just online, but also in person. I was able to learn better online.” (Josefina) “I definitely want to continue doing so, able to use zoom. Dipping my toes in the water.” (Lucas)
Relatedness professional relationships with students	“We have our weekly meetings, and we all have a pretty good relationship. For my students, in person, I had a good rapport.” (Josefina)	“Once the spring happened, I lost of my students who were struggling, and who remained were my higher-level students. We’d go over the stuff.” (Lucas)

**Table 4:** Supporting quotations from LAs’ Questionnaire responses to the three constructs of SDT including lessons learned during and after remote learning transition

SDT construct	Lessons learned during and after remote learning experience
Competence	“Guiding students towards achieving a better grade is a lot more challenging as I initially thought. Students come from different backgrounds with varying levels of assimilation, learning styles and prior knowledge.” (Rashidi) “I have learned that teaching students multiple ways of solving problems is best because not all the same methods will click with every student.” (Josefina) “I have learned that there is an art to asking questions, and that phrasing can change a student’s complete outlook on a question.” (Lucas) “I learned that it is extremely challenging to identify what specific mathematical concepts students may be having trouble with and being able to explain the concept in ways outside of the way that I was originally taught.” (Rachel)
Autonomy	“Upon become a learning assistant, I found that there were multiple subject areas where my learning was actually limited compared to what’s required in my class. Refining these subjects has helped me greatly.” (Dylan) “You can certainly prepare ahead of time, but there is going be some questions, where you have to say “I’ll get back to you on this,” to prevent from spending the entire time on that one question.” (Ramesh) “Explaining to the students what the different subjects will be used for in their daily lives helped me relate it back to myself. For instance, I was explaining how the concept of sequences helps us understand that something can be infinitely small and still increasing, which helps us be more open minded and welcoming to challenges in our daily lives.” (Chloe) “So being able to talk and share mathematics with my students and address multiple ideas and opinions on problems is something I can apply to so many other things.” (Maria)
Relatedness	“This struggle [learning content] yielded the best fruit because we were able to work through the class together as a team instead of a hierarchical dynamic that nobody wants.” (Chloe) “Whether it be through the email and text, chat or speech, perhaps certain people feel more inclined to ask questions live so I learned to allow for multiple ways to reach out to the students.” (Ramesh) “I didn’t anticipate students being so reluctant to ask me questions. I thought since I am student just like them they would have no problem asking for help but that is not always the case.” (Josefina) “Working with different students with different backgrounds, this has thought me to be patient with the students for their grades to be excellent. This is a key virtue that will help in the future.” (Rashidi)

Table 1 shows sampled quotations within each construct of SDT to give the reader an understanding of the lines of demarcation between the three constructs of SDT as conceptualized in this study. Competence was coded as outward displays of being capable to perform their role of LAs, which included improving content knowledge and skills. Autonomy was coded as inward reflections, making decisions, and reflecting on current and future practice as an LA. Relatedness was coded as the personal interactions made between stakeholders (e.g., STEM students, mentor faculty, other LAs). This category took into account personal observations and feelings of connectedness they experienced as LAs. This codebook ensured that each unit of data, produced from the first pass, was categorized into one—and only one—construct of SDT. For example, Josefina conceptualized her competence in how she used tools (Zoom) to perform her job as an LA, whereas Lucas also discussed tools (Zoom) in the context of competence, as he stated he wanted to use Zoom as an LA in the future.

Third and last, frequencies and summaries among the three constructs were tabulated by data type and parsed by pre- and post-transition to remote learning per SDT construct. This strategy was employed because content “codes are most often numeric…[such that] the conclusion of the typical content analysis [provides a] statistical summarization and analysis of the coded variables across many units of analysis” (Neuendorf, 2019, p. 212). This means of presenting SDT findings is recommended by qualitative content methodologists (e.g., Selvi, 2019) and found in concurrent literature exploring students’ experiences by SDT constructs (e.g., Haselberger et al., 2020). By parsing journal entries and focus group data into two distinct time periods (before and just after transition to remote learning, respectively), with a questionnaire as a delayed post measure, we are able to explore changes in LAs’ reports of the three aspects of their self-determination within this period of transition. We utilize quotations (from journal entries in Table 2 and questionnaire data in Table 4) and utterances (from focus group discussion in Table 3) to qualify our SDT-based interpretation of frequency counts and trends in the data. Given that the journal entries were completed by all LAs in the study and contains data both before and after the transition online, that data is presented first and supported by focus group and questionnaire data. By looking across the time periods of interest, leading with journal entries supported by the questionnaire and focus group data, we were able to determine fluctuations in constructs that contribute to self-determination among LAs and assess those impacts.

### Trustworthiness

Given the inherently qualitative nature of the data collected and analyzed, efforts towards trustworthiness (Lincoln & Guba, 1985) were made to ensure rigor in data collection, analysis, and interpretation of results. Credibility was established by use of validated theory (i.e., SDT) with strong concurrent validity in modeling and measuring motivation (e.g., persistence and performance) among teachers and LAs. By collecting various sources of qualitative data (journal entries, focus group data and questionnaires), we are able to show the broader notions of SDT throughout the time period and among the LAs sampled. Using SDT theory constructs as an a priori coding schema aided confirmability as well as strict adherence to the qualitative content analysis method (Krippendorff, 1980). Further, interrater reliability (IRR) was performed with each of the three data sets to affirm the coding process and findings. For the journal data, a second researcher double-coded the data set, determining percent agreement within each of the three categories for each LA. After the first round of coding, percent agreement was averaged among the three constructs, which determined to what extent each unit of data was categorized as relating to SDT and then coded to the same SDT construct by both of the coders. This first round found that coders were 89% accurate for Eduardo, 87% for Rachel, 87% for Chloe, 84% for Alejandra, 81% for Connor, 79% for Maria, 78% for Lucas, 78% for Arturo, 75% for Josefina, 73% for Jake, 68% for Ramesh, 65% for Rashidi, and 46% for Dylan. Overall, the competence construct had lowest agreement at 72% followed by relatedness at 74% agreement, whereas autonomy had 82% agreement. The primary reason for coder disagreements were related to the codebook, with the constructs of competence and autonomy needing greater distinction in their definitions for more accurate coding (which produced Table 1 to aid in fidelity of coding SDT constructs). Further, some LAs like Dylan had little data coded, so any disagreement between coders produced very low values that affected construct-level averages. Once the codebook was revised, student-level data with less than 80% agreement (i.e., all LAs except Alejandra, Chloe, Connor, Eduardo, and Rachel) were reviewed in a second round of coding by a third coder, who attended all conferences and discussions between both coder 1 and 2, independently resolved disagreements and produced the final codes. For questionnaire data, a second coder interrated each of the 87 coded units of data, finding 12 areas of disagreement for an IRR of 86%. Again, the same third coder reviewed independently resolved the 12 disagreements to decide and develop the final code set. For the focus group data, the transcript was read and reviewed by both coder 1 and 2, finding 100% agreement among the codes. The 100% agreement was likely due to the low number of coded data (*n* = 10) provided by the three LAs shared during the focus group.

Notably, dependability and transferability are also included within the Lincoln and Guba (1985) trustworthiness paradigm. However, this research is not meant to describe shifts among all LAs’ self-determination vis-à-vis COVID-19 interruptions to course delivery; the intent is to provide insight to the experiences sampled LAs had in this unique situation. By modeling this situation, we may better understand how their self-determination was impacted. When similar or continued interruptions occur that influence LAs’ self-determination, we may utilize strategies to bolster their motivation and performance in supporting undergraduate STEM students.

## Results

The results of deductive coding from journal entries found that among the total of the 714 coded units of data, 336 (47%) were related to self-determination in F2F instruction: 161 (48%) in the construct of competence, 98 (29%) in the construct of autonomy, and 78 (23%) in the construct of relatedness. Individual students’ responses by constructs with totals are found numerically in Appendix: Table 5 and as bar graphs in Figs. 1, 2. Upon transition, among the 378 (53%) remaining coded units of data collected during remote instruction evidenced 154 (41%) related to the construct of competence, 119 (31%) to the construct of relatedness, and 105 (28%) to the construct of autonomy.

**Table 5:** Frequency count of learning assistant reflections related to the constructs of SDT before and after the remote learning experience

LA Pseudonym	Competence	Relatedness	Autonomy
Rashidi			
Pre	13	19	11
Post	13	17	11
Josefina			
Pre	7	10	3
Post	5	3	1
Jake			
Pre	11	1	11
Post	11	3	4
Lucas			
Pre	11	6	6
Post	8	8	11
Connor			
Pre	5	5	11
Post	15	8	9
Dylan			
Pre	22	5	4
Post	0	0	0
Eduardo			
Pre	16	5	7
Post	15	11	10
Arturo			
Pre	7	5	10
Post	14	7	6
Rachel			
Pre	12	5	7
Post	13	13	15
Ramesh			
Pre	29	10	11
Post	17	8	3
Chloe			
Pre	11	1	6
Post	17	19	12
Maria			
Pre	2	3	3
Post	10	10	10
Alejandra			
Pre	15	3	7
Post	16	12	13
Total			
Pre	161	78	97
Post	154	119	105

### Journal entries

Figure 2 shows the bar graph of LAs’ tallies by individuals. Results show the frequency counts from sampled LAs, sourced from their journal entries, that reported aspects of competence decreased by 7% (from 48 to 41%). The LAs writing about aspects of autonomy decreased by 1% (from 29 to 28%) and relatedness increased by 8% (from 23 to 31%).

*Competence* Upon exploring the data qualitatively, shifts in competence were attributed to LAs being unable to view physical cues from students’ faces or body language, such as when they were confused or uncomfortable with the content, as they had in the F2F setting. Alejandra initially wrote in her journal entry (2-29-2020), “I think every learning assistant can tell what group of students in their row know the content and what students don’t” which was followed later (5-5-20) with “having the ability to physically see the students in my row made it easier to interact and get them to work with each other,” which she attributed to “leav[ing] me the satisfaction of being a helpful source for them.” Ramesh and Eduardo both discussed how they struggled with guiding students towards the answer, rather than just giving them answers since they mentioned having struggled with the content and student interactions, respectively. Maria discussed in her reflections that being a mathematics major directly aided her perceived competence as an LA. Her sense of competence was reinforced while being an LA; she described how her LA experiences increased her content knowledge and skills. Although the modality changed, her ability to learn from her faculty mentors and in Calculus did not, which led her to state she would like to be an LA into the future.

*Autonomy* In regard to autonomy, Chloe had stated on 2-25-20 that her autonomy as an LA sprung from “time adapting to their individual needs…being resilient and flexible.” However, that autonomy shifted when on 5-2-20, she expressed an inward reflection in that, “the level of enthusiasm [has] decreased with the switch to online learning, due to the fact that students were sent home, which is an environment that they usually only go back to for breaks/summers.” Lucas was not certain of his efficacy as an LA, even when the lectures and LA meetings were face to face. When transitioning online, he felt that student accountability had decreased, which made it harder for him to be able to motivate his students in learning Calculus II. He attributed his shift in autonomy to the lack of interaction online, the distractions of working completely online, and ongoing problems with communicating with his STEM students. Jake had a similar experience as he experimented with different teaching strategies as an LA. He found that online teaching was the most challenging; suggesting more time dedicated to lecture pre-transition and post-transition finding effective ways of communication post-transition. He stated he would like to continue as LA in either modality.

*Relatedness* Relatedness was the construct that increased, where many LAs acknowledged the importance of forging and maintaining strong connections online, employing strategies to strengthen connections (Jake, Rachel, Ramesh), taking leadership in teaching to use Zoom tools (Lucas, Maria), and extending the learning environment by use of social media and texting (Chloe, Rashidi). However, some LAs felt more distant (Josephina, Dylan) and removed (Arturo, Lucas) from the undergraduate learners. As examples, Arturo was unsure how to engage students prior to COVID-19; he reflected that his challenge is related to a poverty of relationships with his students overall (i.e., he was not remembering names, not engaging in a group chat like the other LAs, etc.). He noted that going online only made these relationships more strained, finding his students were not engaging with him, causing him to feel his effectiveness as an LA was even worse in the online setting. Josefina had stable relationships with both her students and faculty members, yet she struggled in the transition to online learning, feeling that she became distant from faculty and students in the new modality. She adapted by devising ways to adjust to the video conferencing format to enhance interaction. However, she felt that only a few students interact with her (as compared to before), noting that students felt less comfortable and consequently quieter. Rashidi chose to employ resources given from mentors, first focusing on content and later on developing relationships when the courses migrated online. He credited his success by being cognizant of the importance of students being able to communicate (their struggles during this time), rather than them wanting to communicate (only about Calculus). He found himself becoming very engaged during the transition and felt very connected and satisfied from his experiences in the LA program that term. Given the importance of relatedness, and its connection to both perceptions of both competence and autonomy, LAs began shifting their focus away from competency (focused on conveying content) towards strategies to enhance relatedness given the reported disconnect between themselves and their undergraduate students in the remote environment. This finding is important, given that relatedness is the guiding construct of self-determination (Darner, 2009). We found a similar relationship between relatedness and autonomy with Rachel. She wrote about her dedication to her STEM students, affectionately referring to them as her ‘kiddos.’ She had focused her attention, throughout her LA experience, on how much her students were participating and how comfortable they felt with the shift in modality, even from the very first online lecture. Her strong sense of relatedness was prevalent in her reflections and was inexorably tied to her effective decision-making (autonomy) as an LA. Table 2 provides additional quotes that evidence the shifts in self-determination as described within LAs’ journal entries.

### Focus group discussion

In the focus group data, Table 3 displays quotes aligned to the constructs of SDT before, during and after COVID-19 shared by the three LAs who participated in the focus group interview. Regarding competency, defined as experiences making you feel more competent, Eduardo shared that before COVID-19, “…being able to see [the students] work it out” afforded Eduardo to feel more successful in his role as a LA. After COVID-19, the LAs remarked that they had to use the online video conferencing software capabilities, such as using the whiteboard or breakout room functions, to support feeling more confident in an online environment. Before COVID-19, Lucas shared that he felt less confident as a LA; he questioned his ability to teach the mathematical concepts correctly. However, after COVID-19, Lucas stated that in the online environment, he would often get derailed after switching to the online breakout rooms. When his flow of working with the students was interrupted, Lucas believed that he lost the respect of the students. Relatedness, as defined in this focus group as professional relationships with students, Josefina shared that before COVID-19 she had developed a good relationship and rapport with the students, citing that the weekly in-person meetings were helpful in building and maintaining their relationships. After the transition to online support for students due to COVID-19, Lucas remarked that it was more difficult to support a professional relationship with the students that he perceived needed his assistance the most. He elaborated upon that point, identifying that the students who continued to meet with him during the pandemic were his higher performing students. Lucas recalled that the students who were struggling were not keeping in contact with him. The LAs also provided information regarding autonomy before and after COVID-19 and the transition to an online space to support students. In this context, the autonomy construct was defined as being empowered to make decisions regarding the duties and responsibilities of LAs. Eduardo shared that the LAs were using their own pedagogical methods to review mathematics concepts with students pre-COVID-19, noting that choosing the best way to review information with the students was determined by the LA. After COVID-19, the LAs shared that they continued to choose the best pedagogical approach to review information with students through the use of the available tools, such as online video conferencing software. Josefina noted that using the whiteboard was a specialized tool she used to engage the students in the learning process. Overall, the LAs shared specific instances of competency, relatedness, and autonomy before and after the change to an online platform due to COVID-19. Although there were some issues highlighted by the LAs regarding the use of an online video conference application to support students, the LAs continued to build their competence to teach students, build and sustain relationships with students, and chose specific pedagogies and tools to support student learning.

### Questionnaire responses 

From the questionnaire data, Table 4 depicts selected quotes from LAs sharing lessons learned after the transition to a remote learning environment using online video conferencing tools to support student learning. These experiences related to lessons learned were connected to the constructs of SDT: competency, relatedness, and autonomy. Related to competency, LAs noted the challenge of learning how to support diverse students, realizing that in growing an LA’s competency, acknowledging diversity and supporting students with diverse methods is best to support learning. Josefina shared that she learned to teach a concept in multiple ways to support student learning. Rachel noted that it was challenging to explain mathematical concepts in different ways, and Lucas, cited that “there is an art to asking questions.” Although these students shared the challenges associated with growing their own skills, the LAs reflected on their growth and competency acknowledging that there is not one method to support learning, but there were multiple methods and strategies to teach a concept to a diverse audience. Developing professional relationships, a hallmark of the relatedness construct, was another lesson learned throughout this experience. LAs were surprised that some students were reluctant to ask questions. Josefina believed that “…since I am a student just like them, they would have no problem asking for help, but that is not always the case.” In creating a relationship with students so that they are comfortable in asking questions to the LAs, Ramesh stated, “…I learned to allow for multiple ways to reach out to the students…” and allowed communication to come from many different forms including email, text, chat, or speech. Chloe was adamant that as part of the relationship building process with the students, she ensured that her class worked together “…as a team instead of a hierarchical dynamic that nobody wants.” LAs’ reflections suggest that open communication and the culture of the learning environment are important aspects to support relationship building with learners. Regarding the construct of autonomy, LAs shared that the process of preparing for these sessions and choosing diverse methods to support learning has strengthened how they as LAs would connect or apply these topics to the students’ lives outside of the learning environment with the LAs. Maria noted that she now can apply the context to “…multiple ideas and opinions on problems…” with her students, and in agreement, Chloe stated that “Explaining to the students what the different subjects will be used for in their daily lives helped me relate it back to myself.” Her reflection relates to autonomy as she is making meta-cognitive assessments that not only improve upon her current performance as an LA, but also future performances as an LA (see Table 1 for construct indicators). Maria and Chloe chose to relate specific mathematical concepts to their students’ lived experiences to support learning. Additionally, Dylan and Ramesh shared preparation before the class by choosing specific methods that were crucial to support student learning. Overall, data suggest that over time, they continually reflected and improved on their teaching practices. According to the constructs of SDT, motivation may have been an influential factor in the LAs continuing to participate in the learning process by teaching concepts in multiple ways, acknowledging the power of student diversity, creating open communication with students, relating mathematical concepts to students’ lived experiences, and planning for classes.

### Limitations

Notable limitations include how assessments of self-efficacy were made and the timing of LAs’ written reflections. In this study, we used LAs’ written and oral self-reports to make assessments of their self-determination. Other aspects of their self-determination that were not reported in journal entries, focus group, or the questionnaire may exist, but were not captured that may have contributed to their perceptions of self-determination. Also, LAs would sometimes submit their reflections on the Monday after the due date; other LAs would skip a week, resulting in fewer reflections submitted overall. We have mitigated this limitation by parsing and examining data in two time points (before and after remote learning transition), rather than week-by-week. Another limitation is that LA weekly responses were sent directly to the two cooperating faculty members. Because LAs knew that their responses were being read by faculty and researchers alike, LAs may have muted or modified their responses due to the Hawthorne effect. Because writing and submitting reflections directly to faculty members has been a part of the culture of all LA programs and in this university program (see LAA, 2020d), sampled LAs were unmotivated to provide incomplete or inconsistent information. This assumption was affirmed by the consistency in the content and focus of reflections analyzed throughout the study and among the focus group responses, in which faculty were not present or privy. Another limitation is that prompts were more focused during and after the transition period, which may have led to more salient examples of competency, autonomy, and relatedness. A last, but notable limitation, was that these LAs served students in Calculus II, which a difficult undergraduate course for most STEM students as evidenced by the high failure rates and attrition (Peck et al., 2016), especially for students from under-represented groups in STEM (Sanabria & Penner, 2017). This may have provided LAs with additional challenges feeling self-determined in supporting these struggling students successfully. Having baseline data (written reflections) from the start of the course helps to visualize challenges to sampled LAs’ self-determination within the context of the course, such that we are comparing ‘apples to apples.’ However, great caution should be made when comparing the results of this study to all LAs’ experiences serving during the pandemic given the great variation within LA contexts (e.g., quality of training and faculty mentoring, type of STEM course they co-instruct, etc.). For example, some LA programs may have chosen to reduce class sizes for social distancing and mandatory face coverings to preserve in-person instruction. However, it seems at this time that many universities were transitioning LA’s work to online modalities (Emenike et al., 2020).

## Discussion

As near peers, students who are motivated to volunteer and serve as LAs forge strong relationships among undergraduate STEM students, which have been found to be the foundational affective structures within successful student-support interventions (Jardine, 2020; Li, 2013). Therefore, investigating how the rapid shift in course delivery, due to the pandemic, impacted LAs’ self-determination (motivation) to serve undergraduate STEM students can help inform efforts to mitigate future, drastic changes to LA support and course changes and prepare to better absorb the blow of future shocks. By using SDT per Ryan and Deci (2000a, 2000b, 2002), we were able to empirically explore how COVID-19 interruptions to course delivery have impacted LAs’ self-determination through the constructs of competency, autonomy, and relatedness. We found that LAs reflected proportionally less on competency and autonomy, but more on relatedness, after the transition to remote instruction.

Regarding the decrease in competency, these results suggest that without the F2F or direct interaction with students, LAs’ perceptions of their competency faltered, despite having a strong background in Calculus. Their writing revealed that many relied on physical clues (e.g., reading students’ faces for signs of frustration) and assistance (e.g., drawing models on paper) to gauge students’ ideas and how they were constructing knowledge, which was markedly reduced in the online modality. Over time, LAs turned to language to elicit students’ comprehension of the material, asking students to describe their mental models or show (to the web camera) their thinking in smaller virtual break-out rooms. This rapid and unexpected shift in their pedagogical approach was a commonly cited frustration among LAs’ ability to serve their students. Research has suggested that when LAs are presented with environments unlike how they learned STEM content and possess limited experiences utilizing novel pedagogies, they are likely to struggle with reconciling their pre-conceived notions of teaching and implementing new instructional techniques (Top et al., 2018). Given that they were learning online pedagogies in real time, with reduced interaction with students and faculty mentors, these challenges became even greater.

While not as substantial a decrease as competence, results suggest a slight proportional decrease in the frequency of LAs’ references to their sense of autonomy. We contend that this lesser attention to autonomy was due to LAs’ focused attention on ensuring that minimal learning objectives were at least being met, considering the arduous task of teaching Calculus in a virtual learning environment without prior training. Ali (2020) asserted that a successful transition to remote learning requires the willingness to embrace change. LAs, perhaps like many other in-person instructors, felt ill-equipped to assist learners completely online and were perhaps hoping for more guidance instead of seeking interventions driven autonomously. Despite the proportional decrease in frequency of LA reports in reference to the construct of autonomy, LAs still began to engage in new behaviors, such as to take more control over the learning environment. We saw LAs begin to employ new online teaching tools (Maria, Lucas) and foster online communities (Chloe, Rashidi) as means for additional support within the online and remote learning modality. Nine of the 13 LAs self-engaged in some type of autonomous behavior focused on improving their communication and access to students. Naturally, we believe that through those actions, LAs had enhanced their connections to students, especially in how they forged new relationships with the students they served.

The study’s key findings were in relation to the COVID-19 transition impacts on LAs' relatedness, suggesting that students became more invested in developing relationships online in order to better support these learners. Findings are affirmed from a concurrent study for more empathetic and inclusive pedagogies employed in online courses (Rapanta et al., 2020). Furthermore, these findings suggest relatedness was specifically focused on relationships between the LAs and the undergraduate student/s. Given the importance of the relatedness construct to one’s overall self-determination, LAs struggled to connect with their assigned students when they transitioned to remote learning, which may explain the increase in their reports of relatedness issues in their journal writings. There is growing appreciation of the power of relatedness between LAs (and other near peers) and the success of undergraduate STEM students. A study by Winterton et al. (2020) found that in large introductory STEM courses the undergraduate students’ learning gains were greatest among those who best related to their near peer, more than any other factor measured. Moreover, SDT research suggests that among college students (compared to pre-college students), relatedness is the most salient factor in their STEM learning (Trenshaw et al., 2016). This research showed how LAs restored their self-determination despite having early dips in competency, which fostered a rise in autonomous actions to foster relatedness among students and each other to maintain their motivation as LAs. Our research suggests that relatedness (i.e., the importance of and strategies in) should lead rather than lag in LA preparation, to ensure sustained and consistent motivation of LAs as they engage in their important work.

## Conclusions

The three constructs of SDT, competence, autonomy, and relatedness provide an ideal framework from which to model motivation among LAs in undergraduate STEM courses. Employing such a model is important to measuring and monitoring LA self-determination for their persistence and performance in serving undergraduate STEM students. With the interruption of COVID-19, moving courses from F2F to online, remote instruction permits a novel understanding to how increasing interruptions influence LAs’ motivation and perceptions of how they were able to assist undergraduate STEM learners. We found that the precipitous drop in their perceived ability to help their students (competence) drew LAs to conceptualize and enact ways to reconfigure their interactions in the course (autonomy). Such efforts not only aided them in reclaiming their competence, but also fostered new avenues of relatedness, which they found to be incredibly important for their students’ learning of Calculus and keeping them connected to the course.

This research suggests new areas of professional development for LAs to strengthen relationships between themselves, their mentors and undergraduates STEM students as they continue to serve in the new and quasi-permanent employment of being an ‘online’ LA. To ensure recruitment and retainment of motivated LAs, we recommend specific strategies in: recruitment (revising LA selection standards beyond content knowledge (competence) to include autonomy and relatedness aspects); clarification of their role (specifically for remote spaces to enhance LA autonomy), new methods of mentoring (modeling how to engage in relatedness when online), and training in best practices for online instruction. We suggest that this research begins new actions and research exploring how to train LAs in strategies to strengthen relatedness, so they are better able (motivationally) to serve STEM undergraduate students. Considering the role of external forces on LA’s self-determination (like the transition from F2F instruction to remote course delivery), recommendations for future research include examining how LAs’ self-determination is influenced at scale, such as the programmatic (faculty mentors) and university level (policy changes).

## Data Availability

The datasets used and/or analyzed during the current study are available from the corresponding author on reasonable request.

## Appendix

See Table 5.

## Abbreviations

COVID-19: Coronavirus disease of 2019
F2F: Face-to-face
IRB: Institutional review board
IRR: Interrater reliability
LAs: Learning assistants
LAA: Learning assistance alliance
SDT: Self-determination theory
STEM: Science, technology, engineering, and mathematics
